# Multiscale Characterization and Biomimetic Design of Porcupine Quills for Enhanced Mechanical Performance

**DOI:** 10.3390/ma17091949

**Published:** 2024-04-23

**Authors:** Lili Liu, Yurong Wang, Jianyong Zhao, Zhihao Cai, Ce Guo, Longhai Li

**Affiliations:** 1 School of Mechanical and Electrical Engineering, Xuzhou University of Technology, Xuzhou 221018, China; 11326@xzit.edu.cn (L.L.); 20211015201@xzit.edu.cn (Y.W.); 20200602149@xzit.edu.cn (Z.C.); 2 College of Electrical Engineering, Zhejiang University, Zheda Road 38, Hangzhou 310027, China; jyzhao@zju.edu.cn; 3 Institute of Bio-inspired Structure and Surface Engineering, Nanjing University of Aeronautics and Astronautics, 29 Yudao Street, Nanjing 210016, China; guozc@nuaa.edu.cn

**Keywords:** porcupine quills, nanomechanical, macro-mechanical properties, compression, torsion, impact performance

## Abstract

The mechanical properties of porcupine quills have attracted the interest of researchers due to their unique structure and composition. However, there is still a knowledge gap in understanding how these properties can be utilized to design biomimetic structures with enhanced performance. This study delves into the nanomechanical and macro-mechanical properties of porcupine quills, unveiling varied elastic moduli across different regions and cross sections. The results indicated that the elastic moduli of the upper and lower epidermis were higher at 8.13 ± 0.05 GPa and 7.71 ± 0.14 GPa, respectively, compared to other regions. In contrast, the elastic modulus of the mid-dermis of the quill mid-section was measured to be 7.16 ± 0.10 GPa. Based on the micro- and macro-structural analysis of porcupine quills, which revealed distinct variations in elastic moduli across different regions and cross sections, various biomimetic porous structures (BPSs) were designed. These BPSs were inspired by the unique properties of the quills and aimed to replicate and enhance their mechanical characteristics in engineering applications. Compression, torsion, and impact tests illustrated the efficacy of structures with filled hexagons and circles in improving performance. This study showed enhancements in maximum torsional load and crashworthiness with an increase in filled structures. Particularly noteworthy was the biomimetic porous circular structure 3 (BPCS_3), which displayed exceptional achievements in average energy absorption (28.37 J) and specific energy absorption (919.82 J/kg). Finally, a response surface-based optimization method is proposed to enhance the design of the structure under combined compression-torsion loads, with the goal of reducing mass and deformation. This research contributes to the field of biomimetics by exploring the potential applications of porcupine quill-inspired structures in fields such as robotics, drive shafts, and aerospace engineering.

## 1. Introduction

Biomimetic structures, inspired by natural designs found in living organisms, have gained popularity in engineering and design. For example, the tube-like structure, known for its excellent mechanical strength and high energy absorption capacity, can be produced cost-effectively [1,2]. These structures, which have evolved over billions of years, offer optimized mechanical and multi-functional properties that are highly desirable across industries such as aerospace, automotive, and military [3,4,5]. Through natural selection, creatures have developed structures with properties like impact resistance, fracture resistance, and lightness, making them ideal for various design applications [6,7]. By drawing inspiration from nature, designers can tap into innovative ideas that leverage the exceptional properties exhibited by natural structures [8]. Inspired by nature, researchers have explored the design of innovative structures based on biological principles. Wei et al. developed thin-walled structures mimicking hedgehog spines and beetle forewings to improve deformation coordination and stress distribution [9]. Niu et al. enhanced honeycomb structures by extracting the beetle elytra intersection unit, leading to improved crashworthiness in bionic honeycombs [10]. Liang et al. optimized bionic shrimp chela multi-cell tubes for energy absorption in bending, demonstrating superior crashworthiness [11]. Song et al. examined the energy absorption efficiency of bionic conch structures compared to conventional tubes, showcasing higher performance in crashworthiness design [12]. Zhou et al. proposed a bionic tube inspired by yak horn for enhanced energy absorption, outperforming traditional tube designs [13]. Cui et al. designed hierarchical lattice structures with cuttlebone-like features, showing excellent mechanical properties and specific energy absorption [14]. Pezhman et al. studied the energy absorption characteristics of bamboo-inspired bionic structures, highlighting the importance of rate-dependent material models for impact modeling [15]. Wang et al. introduced a novel multi-cell tubular structure inspired by the glass sponge skeleton, demonstrating superior mechanical properties and energy absorption capacity [16]. Wei et al. designed a size-gradient thin-walled structure mimicking an impact-resistant antler, showing exceptional crashworthiness performance with high specific energy absorption and efficiency [17].

Researchers have shown that biomimetic structures offer superior strength-to-weight ratios compared to traditional materials [18,19], making them crucial in modern industry to meet the demand for efficient and cost-effective solutions [20,21]. Drawing inspiration from nature, researchers have designed structures for enhanced energy absorption through improved deformation coordination and stress distribution. For example, Song et al. developed a bionic column with grooves based on cornstalk attributes, showing better crash performance than circular columns under lateral loading [22]. Fu et al. studied the energy absorption of a novel bionic bamboo tube structure during axial crushing. Six different cross-sectional configurations were developed, and numerical results showed that BBT structures with specific rib shapes demonstrated improved crashworthiness [23]. Hu et al. created a bionic multi-cell tube with secondary ribs inspired by leaf veins, showing increased energy absorption and mean crushing force compared to traditional tubes [24]. Li et al. fabricated a bio-inspired multicell tube mimicking the glass sponge unit structure, demonstrating high specific energy absorption and outperforming conventional multi-cell tubes [25]. Liang et al. introduced bionic bamboo tubes with enhanced energy absorption potential, particularly in large-angle cases [26]. Lastly, Liu et al. proposed a novel bionic tube design inspired by bamboo vascular bundles, achieving optimal energy absorption through specific structural characteristics [27].

The porcupine quill is primarily characterized by its sharpness and spikiness, providing formidable armor that serves as its primary defense mechanism against potential predators [28,29]. The quills’ unique mechanical and structural features make them an outstanding material for biomimetic design. The smooth surface of the porcupine quill helps reduce resistance and friction during penetration into the ground or other objects. Their conical design provides additional stability and support, particularly important when walking or climbing. Moreover, the quills exhibit high impact resistance due to their internal fiber structure arranged in concentric circles and interlaced in different directions, forming a very strong mesh structure that can effectively absorb and disperse external impact forces, thus ensuring the porcupine’s safety and health when attacked or falling [30]. Furthermore, the conical design of porcupine quills also serves as a defense mechanism. When threatened, porcupines can contract their bodies to make their quills protrude outwards and rapidly rotate them at high speed to increase attack force, quickly driving away or repelling enemies and protecting their lives. Therefore, the exceptional mechanical properties and structural characteristics of the porcupine quill make it an excellent biomimetic template. It exhibits good mechanical performance and impact resistance and functions as a defensive tool, making it an ideal design model for various applications. It is believed that porcupine quills can be considered a remarkable biomimetic template because of their extraordinary mechanical properties and versatility, which have been honed over time to fulfill particular requirements. Through the investigation of these optimized structures, innovative materials and structures can be developed that demonstrate superior performance and enhanced functionality. Biomimetic materials inspired by porcupine quills possess distinctive structures and properties, providing novel solutions with exceptional protective performance and impact resistance for applications. These materials are specifically designed to enhance the penetrative capabilities of medical devices, boost the mechanical strength of aircraft structures, offer energy absorption features in textile manufacturing, optimizing the design of robotic arms and transmission shafts, demonstrating a wide range of potential applications. By integrating materials and structures inspired by porcupine quills, improvements in functionality and efficiency can be realized across different industries, delivering significant value to advancements in scientific and technological development and industrial progress.

In this study, the micro- and macrostructures, mechanical properties, and nanomechanical properties of porcupine quills were investigated. Results showed that porcupine quills have excellent mechanical properties, high specific stiffness, and specific strength. Based on the micro- and macrostructures of porcupine quills, a series of biomimetic porous structures (BPSs) were designed. The finite element method was used to investigate the static comprehensive mechanical behaviors of BPSs. To validate the simulation results, SLA technology with R4600 resin material was used to fabricate the proposed structures. The mechanical properties of different BPSs such as compression and torsion properties were studied and compared with the simulation results. Additionally, the impact of BPSs on crashworthiness performance was discussed. The significance of biomimetic tubing arises from its ability to facilitate the learning process from nature’s designs and to apply them in creating more efficient and effective systems for a variety of applications. By imitating the structure and functions of biological tubes, it is possible to enhance fields such as fluid transportation, materials science, and medical devices.

## 2. Materials and Methods

### 2.1. Microstructural Observation and Biomimetic Design

**Figure 1:**
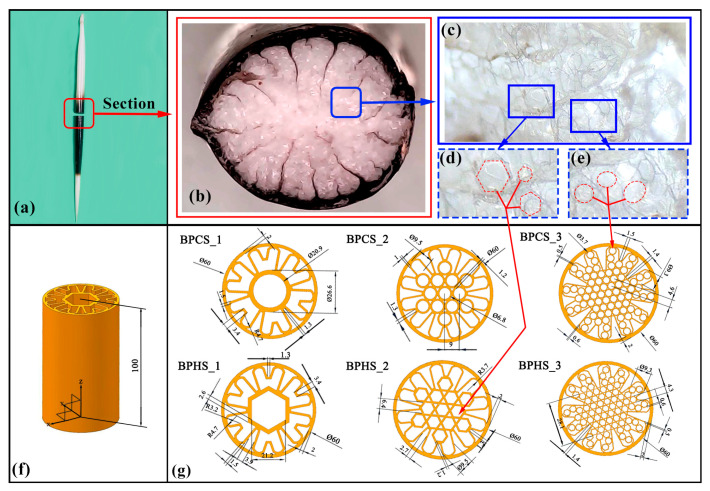
Designed models of bio-inspired structures inspired by porcupine quill. (**a**) Porcupine quill; (**b**) cross section, while panel (**c**) presents an enlarged view; various details of the structure are highlighted in panels (**d**,**e**), which show the portion indicated by arrows in the previous panel. Panels (**f**,**g**) illustrate the various shapes of the thin-walled tubes utilized in the designs.

The porcupine quill was chosen as a biomimetic element for pipe design due to its exceptional mechanical properties, unique microstructure, and impact resistance. The tightly packed and multilayered microstructure, along with its complex morphology, contribute to the quill’s advantageous features. It possesses excellent hardness, strength, toughness, and elasticity, allowing it to withstand high pressure, resist tension, and endure external impacts and deformations. The irregular cell arrangement within the quill enhances its impact resistance by enabling effective distribution and absorption of impact forces. Additionally, the presence of a porous microstructure in the quill allows for cushioning and energy dissipation during impacts, contributing to its overall resilience. These features can be incorporated into pipe design to enhance their ability to withstand sudden impacts and provide increased resistance to damage. The porcupine quill’s outstanding mechanical properties and impact resistance make it a valuable source of inspiration for improving pipe design. Figure 1a depicts a porcupine quill, while (b) illustrates its cross section; (c)~(e) show its microstructure, which consists of circular and irregular polygons.

The selection of these sets of designs is primarily based on the internal microstructure of porcupine quills and has been improved and optimized through different filling methods and sizes. In the first set of designs, a small circle is designed in the hollow center of the pipe, with petal-like shapes surrounding it. Three variations were considered: the first design incorporated a steel frame in the shape of petals, the second design filled the inside of the petals with small circles of the same size, and the third design used smaller circles to fill the inside of the petals. These variations aim to explore the effects of different types and sizes of filling on the mechanical properties and application performance of the pipe. Similarly, the second set of designs is based on the hollow center of the pipe but incorporates a hexagonal-shaped steel frame, with petal-like shapes surrounding it. The three variations include a steel frame in the shape of a hexagon, filling the inside of the petals with small hexagons of the same size, and using smaller hexagons to fill the inside of the petals. These variations aim to compare the effects of different internal steel frames and fillings on the mechanical properties of the pipe. Through testing experiments and simulations involving compression, torsion, and impact, these six pipes were subjected to various tests in order to compare their mechanical properties and application performance. These experiments provide scientific evidence and further improvements for biomimetic pipeline designs based on porcupine quill structures. Driven by the above promising findings, according to the special structural characteristics of porcupine quill, a series of biomimetic porous hexagonal structures (BPHSs) and biomimetic porous circular structures (BPCSs) were proposed, as shown in Figure 1f,g. The selection of these sets of designs is based on the internal microstructure of porcupine quills and has been improved and optimized through different filling methods and sizes. This study aims to compare the mechanical properties and performance of different patterned pipes for industries like robotic arms, drive shafts, and aerospace. Findings from this research will guide the development of high-performance pipes that prioritize weight reduction while maintaining mechanical requirements. By optimizing pipe properties, more reliable and efficient solutions can be offered for these industries, enhancing equipment efficiency while reducing energy consumption and costs.

### 2.2. Three-Dimensional Printing BPSs

**Figure 2:**
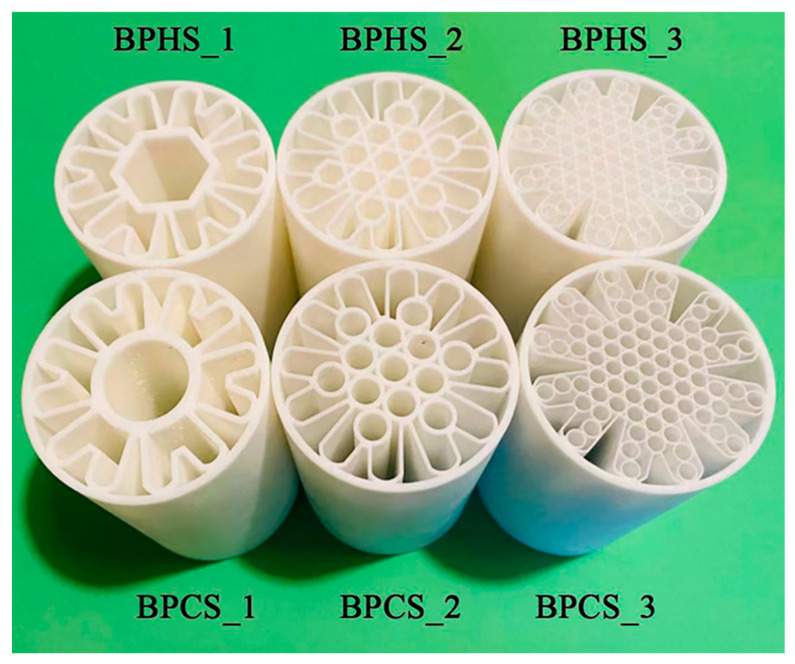
Additively manufactured tubes.

To examine the mechanical properties of the BPSs, simulations were conducted using ANSYS 2021 R1 software. Different loading conditions such as compression and torsion were applied during the simulations. Three-dimensional printing was performed using the LianTai 3D Lite600 printer (Shenzhen, China), which utilizes stereolithography apparatus (SLA) technology. The printer has a print platform size of 800 × 800 × 600 mm and can achieve layer thicknesses ranging from 0.05 to 0.25 mm. It offers high positioning accuracy of ±0.008 mm per layer and excellent printing precision, with a tolerance of ±0.1 mm for prints measuring up to 100 mm, and even higher for larger prints. R4600 resin material was used for manufacturing the actual 3D-printed samples, and its material properties, as provided by the manufacturer, were utilized in all simulations. Table 1 presents the material properties of R4600 resin. Figure 2 displays the tubes that were additively manufactured.

Lightweight number (LWN) is a parameter utilized to assess the efficiency of lightweight design in each BPS. It is calculated as follows:

(1) $$\mathrm{LWN}=\frac{\mathrm{Maxload}}{\mathrm{Weight}}$$

Here, LWN-C represents the lightweight number for compression, and LWN-T represents the lightweight number for torsion. 

### 2.3. Nanoindentation

Nanoindentation is a widely used technique to study the mechanical properties of materials at the micro- and nano-scale. Among various methods to calculate the hardness and elastic modulus from the load–displacement data obtained during nanoindentation tests, Oliver–Pharr theory is one of the most frequently used methods due to its simplicity and versatility. It provides a quick and non-destructive way to evaluate the mechanical properties of small volumes of materials, which is particularly important in the development and characterization of novel materials for various applications in fields such as microelectronics, biomaterials, and nanotechnology. 

The Oliver–Pharr theory states that the hardness and elastic modulus of a material can be determined through nanoindentation testing, which involves applying a small force to a surface using a sharp probe and measuring the resulting indentation depth. By analyzing the relationship between the applied force and the depth of the indentation, it is possible to calculate both the hardness and the elastic modulus of the material. The nanoindentation tests in this study utilized a diamond Berkovich probe with force and displacement resolutions of 10 mN and 0.1 nm, respectively. Maximum indentation depths were kept below 1000 nm, and an unload strain rate of 0.5 (1/s) was applied. The testing was conducted under load control. The experimental parameters consisted of a thermal drift rate of 0.15, an ambient temperature of 25 °C, and a humidity of 70%. The Oliver–Pharr theory was employed to analyze the data obtained from the experiments.

The hardness *H* can be calculated from the following equation:

(2) $$H=\frac{P_{\max}}{A_{c}}$$

where the *P*_max_ represents the maximum load, and *A_c_* represents the contact area.

The elastic modulus *Er* and reduced modulus Er* can be calculated as follows:

(3) $$E_{r}=\frac{\pi }{1-\upsilon^{2}}\frac{S}{2\sqrt{R}}$$

(4) $$E_{r}^{*}=\frac{E}{\left(1-\upsilon^{2}\right)}$$

where *E* is the elastic modulus and *ν* is the Poisson’s ratio. *S* is the contact stiffness, which can be expressed as a function of the unloading stiffness *S_u_*, the displacement *d*, and the radius of curvature *R* of the plastic zone:

(5) S=Su1−dhc

The contact depth *h_c_*, which is related to the radius of curvature *R* of the plastic zone, which is related to the radius of curvature *R* of the plastic zone, can be expressed as follows:

(6) hc=2R/312

### 2.4. Experimental Setup

Finite element simulation was utilized to analyze the mechanical properties of porcupine quills and their bionic structures under various load conditions. To ensure the accuracy of the simulation results, 3D printing technology was implemented to fabricate the proposed structures. Subsequently, compression and torsion tests were conducted using an Instron-5566 universal testing machine and a CTT500 microcomputer-controlled electronic torsion test machine, respectively. During the compression tests, force–displacement curves were obtained at a loading displacement rate of 2 mm/min. The CMT4503 electronic universal testing machine was used for the porcupine quill stretching experiment. Furthermore, the crashworthiness behaviors of the proposed structures were assessed utilizing a drop impact tester (PIT452D-2, Shenzhen Wance, Shenzhen, China).

## 3. Results and Discussion

### 3.1. Nanoproperties

The mechanical properties (elastic modulus and hardness) of distinct regions within porcupine quill, such as the upper epidermis, upper cross section, mid-dermis, mid-cross section, lower epidermis, and lower cross section, were investigated in this study using a commercially available nanoindenter (Agilent Nano Indenter G200, Agilent Technologies, Santa Clara, CA, USA). The depth of the indentation for each sample was carefully controlled to ensure that it was within the elastic deformation regime, thus avoiding any plastic deformation or damage to the sample. Moreover, to prevent any possible deviation from the linear elastic behavior, the maximum indentation depths were kept sufficiently low and well below the critical thickness of the surface layer of the tested samples. The instrument provided precision stages for automated testing and precise positioning of samples. 

**Figure 3:**
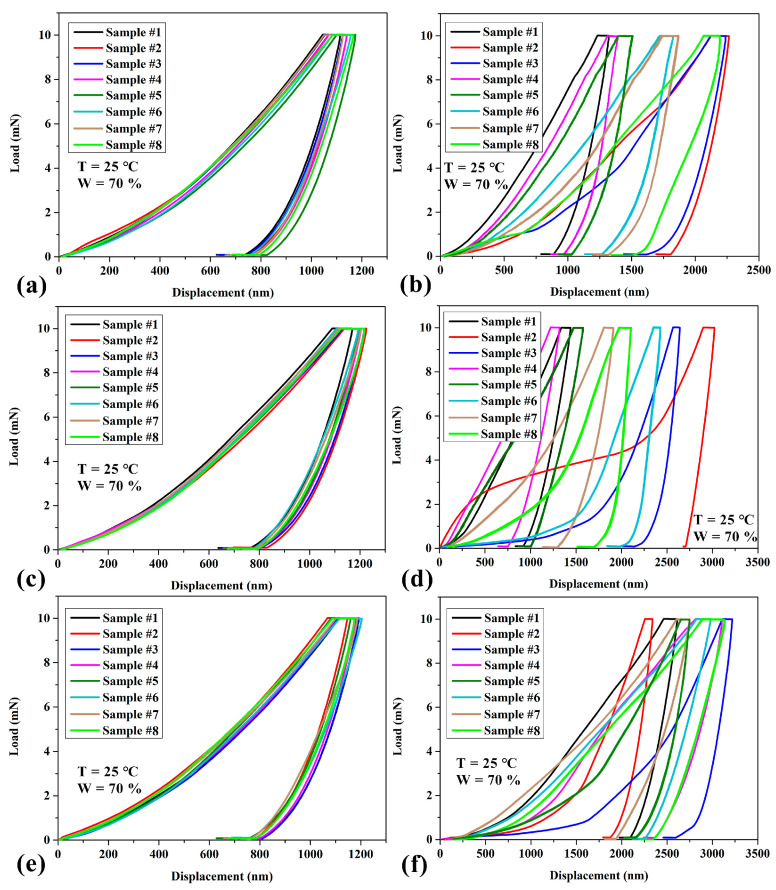
Load–displacement curves of the different regions in porcupine quill. (**a**) Upper epidermis; (**b**) upper cross section; (**c**) mid-dermis; (**d**) mid-cross section; (**e**) lower epidermis; (**f**) lower cross section (including thermal drift section and stable section). Note that the thermal drift section may be attributed to the effect of the surroundings, which can cause instability of the instrument.

Figure 3 shows the load–displacement curves of the different regions in the porcupine quill. Based on experimental data (Figure 3), significant differences were observed in the elastic modulus and hardness among different regions of porcupine quills (Table 2 and Table 3). Table 2 shows the upper and lower epidermis exhibited higher elastic moduli (8.13 ± 0.05 GPa and 7.71 ± 0.14 GPa, respectively) compared to other regions, while the mid-dermis displayed an intermediate value (7.16 ± 0.10 GPa). Furthermore, the elastic modulus at the center of each cross section was relatively lower than that of the surrounding area, with the upper cross section measuring 3.93 ± 0.86 GPa, the mid-cross section measuring 3.48 ± 0.46 GPa, and the lower cross section measuring 2.31 ± 0.71 GPa. It is worth noting that the variations in the elastic modulus among different regions of the quill may be attributed to its unique structure gradient in the vertical direction. Interestingly, the elastic modulus of the mid-cross section fell between the upper and lower epidermis, indicating a gradual change in structure and mechanical properties.

In terms of hardness, Table 3 shows the upper and mid-epidermis demonstrated higher values (0.38 ± 0.02 GPa and 0.34 ± 0.01 GPa, respectively) than the lower epidermis (0.36 ± 0.01 GPa), which is consistent with the trend of elastic modulus. Additionally, there were significant differences in hardness among different regions of the quill, with the upper cross section measuring 0.16 ± 0.09 GPa, the mid-cross section measuring 0.16 ± 0.12 GPa, and the lower cross section measuring 0.06 ± 0.01 GPa. These variations in hardness among different regions may be related to their biological environments and functional needs. The high elastic modulus and hardness values of the porcupine quill make it an effective mechanism for protecting animals against predators and environmental hazards. The quill’s flexibility and elasticity allow it to adapt to the movements and changes of animal bodies while maintaining stability and strength. The distinct structure gradient and multi-layered structure of the porcupine quill have a significant impact on its mechanical properties, which are closely related to its biological functions and ecological environments.

### 3.2. Macroscopic Mechanical Properties

**Figure 4:**
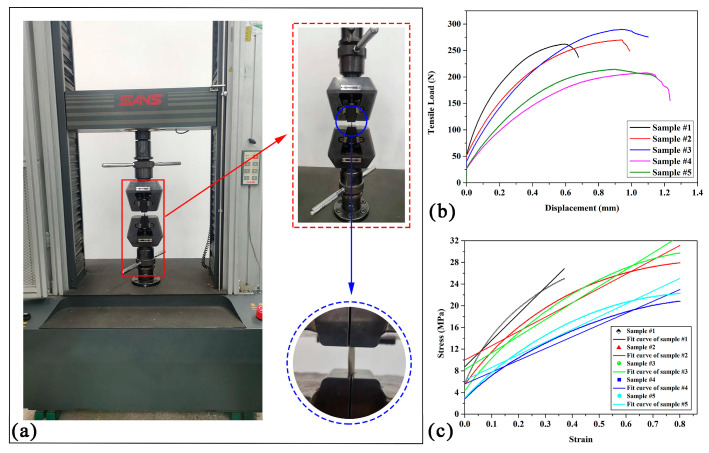
CMT4503 electronic universal testing machine. (**a**) Stretching fixture; (**b**) load–displacement curves of different samples; (**c**) stress–strain curves.

By conducting a tensile test on porcupine quills using a CMT4503 electronic universal testing machine (Figure 4a) with a loading displacement rate of 1 mm/min, a force–displacement curve was generated (Figure 4b). The results obtained from the test indicate that the porcupine quills were subjected to tensile forces until they reached their breaking point. The tensile strength was determined to be 248.95 ± 35.88 N. The data obtained from the test can be used to analyze the mechanical properties of the quills and to gain insights into their strength and durability.

In order to determine the elastic modulus of porcupine quills, a simple linear regression analysis was performed on stress–strain data obtained from five sets of tensile tests. The equation used for the regression analysis was *y* = *kx* + *b*, where y represents the stress values and *x* represents the corresponding strain values. The slope of the line (k) represents the elastic modulus of the material, while the *y*-intercept (b) represents the stress at zero strain. The fitted curves and the corresponding data are presented in Figure 4c and Table 4, respectively.

Table 5 presents the results of five different samples fitted to a linear model *y* = *kx* + *b*. The table includes the sum of squares, mean square, F value, and probability value (Prob > F) for each sample. The F value and Prob > F columns indicate whether the sample is significant or not, where a low probability value (less than 0.05) indicates that the sample is significant. In this case, all five samples have a probability value of 0, which means they are highly significant. Moreover, the high F values suggest that the fit of the linear model is good. The sum of squares column indicates how much of the total variability in the data can be explained by the model, with higher values indicating a better fit. Therefore, based on these results, we can conclude that the linear model *y* = *kx* + *b* is a good fit for the data. The average slope of the fitted curves for the five different samples was calculated, and the variance was determined to obtain its modulus of elasticity result of 30.28 ± 10.98 MPa. The tensile strength of porcupine quills is indicated as 248.95 ± 35.88 N in Figure 4b.

### 3.3. Compressive Properties

**Figure 5:**
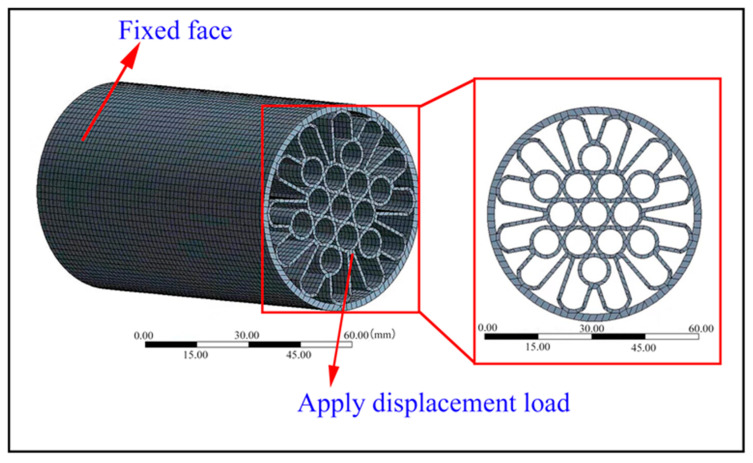
The compression finite element model of BPCS_2.

The compressive properties of the proposed structures were investigated through both experimental and finite element analysis (FEA) methods. Different structures underwent quasi-static compression on their bottom surface while a downward displacement-controlled loading was applied on their upper end along the central axis. Meanwhile, grid convergence tests were conducted to obtain maximum compressive loads and force–displacement curves. In order to ensure FEA accuracy, a simulation using a hexahedral element (Solid186) of 2 mm size with 200,557 nodes and 33,814 elements was carried out. Figure 5 illustrates the compression finite element model for BPCS_2 as an example.

**Figure 6:**
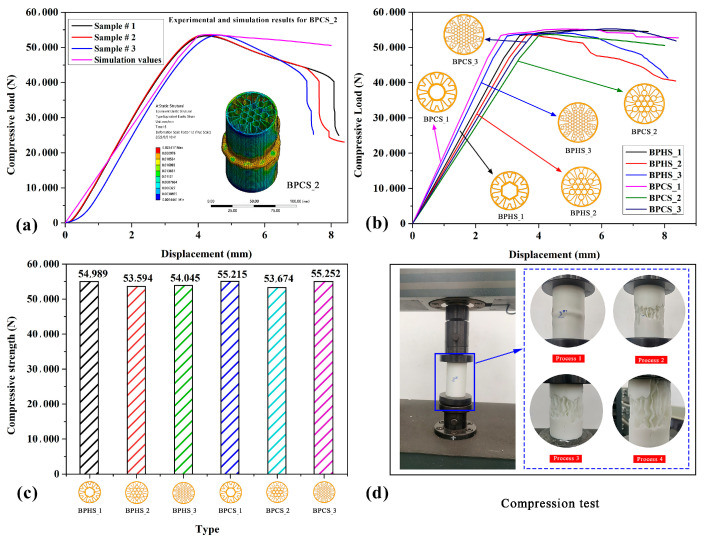
Compressive properties of the proposed BPSs. (**a**) Comparison between experimental results and simulation results for BPCS_2; (**b**) load–displacement simulation curves for different BPSs; (**c**) the maximum compressive load of BPSs; (**d**) compression test.

Figure 6 clearly shows the compression characteristics of the proposed BPSs. In Figure 6a, the experimental results of BPCS_3 are compared with the simulation results. It can be observed from the graph that the maximum compression load of the simulated tube and the three sample tubes follow the same trend, increasing with displacement until reaching a maximum value at around 4 mm displacement, after which the tubes start to fail and the maximum compression load gradually decreases. Additionally, according to Table 6, the tensile strength value of the experimental data is 53.44 ± 0.14 kN, with an error of about 0.43% compared to the simulation data. This result indicates that the simulation results are consistent with the experimental results, and the finite element model can effectively and accurately reflect the experimental data for further research. Figure 6b displays the simulated compression characteristic curves of different BPSs. It can be observed that their compression characteristic curves follow the same trend, with all BPSs’ maximum compression loads increasing with displacement and failing when the maximum compression load is reached. It is evident that the maximum compression loads of different BPSs are relatively close, indicating their similar compression characteristics. More detailed maximum compression load data can be found in Figure 6c. Figure 6c clearly shows the maximum compression load of different BPSs. Firstly, comparing the maximum compression loads of BPHS_1, BPHS_2, and BPHS_3, BPHS_1 has the highest maximum compression load of 54.99 kN, which is 2.61% and 1.74% higher than BPHS_2 and BPHS_3, respectively. This indicates that the number of hexagonal structures filling has a small influence on the compression performance of the thin tube. Secondly, comparing the maximum compression load of BPCS_1, BPCS_2, and BPCS_3, BPCS_2 has a maximum compression load of 53.67 kN, which is 2.89% and 2.94% lower than BPCS_1 and BPCS_3, respectively. The maximum compression load of BPCS_1 and BPCS_3 are close, with BPCS_3’s maximum compression load being 0.05% higher than BPCS_1. This indicates that increasing the number of circular structures filling has a small influence on the compression performance of the thin tube. Finally, comparing BPHS_1 and BPCS_1, BPCS_1 has a maximum compression load 0.42% higher than BPHS_1. Comparing BPHS_2 and BPCS_2, BPCS_2 has a maximum compression load 0.15% higher than BPHS_2. Comparing BPHS_3 and BPCS_3, BPCS_3 has a maximum compression load of 2.22% higher than BPHS_3. This suggests that filling circular structures in thin tubes has a better effect on compression performance than filling hexagonal structures. Figure 6d shows the actual compression test results, where the thin tube was ultimately crushed and produced a large wrinkle after compression.

In addition, Table 6 presents the compressed data from both the experiment and simulation. Upon comparison, it is evident that the maximum compressive load values for both BPHSs and BPCSs are quite similar, as are their corresponding LWN-C values. Notably, BPHS_2 exhibits the lowest LWN-C value of 433.19 (N/g), while BPCS_3 has the highest LWN-C value of 446.61 (N/g). The difference between these two values is only 3.10%, indicating that the compression performance of the thin tube is consistent across the board.

### 3.4. Torsion Properties

The structures were restrained at one end with six degrees of freedom and subjected to angular displacement at the opposite end in order to induce torsion. The reaction torque exerted on the terminal surface was kept constant to determine the torsional load corresponding to the applied torsion angle. The number of grid nodes and elements employed in the torsion simulation matches that used in the compression simulation.

**Figure 7:**
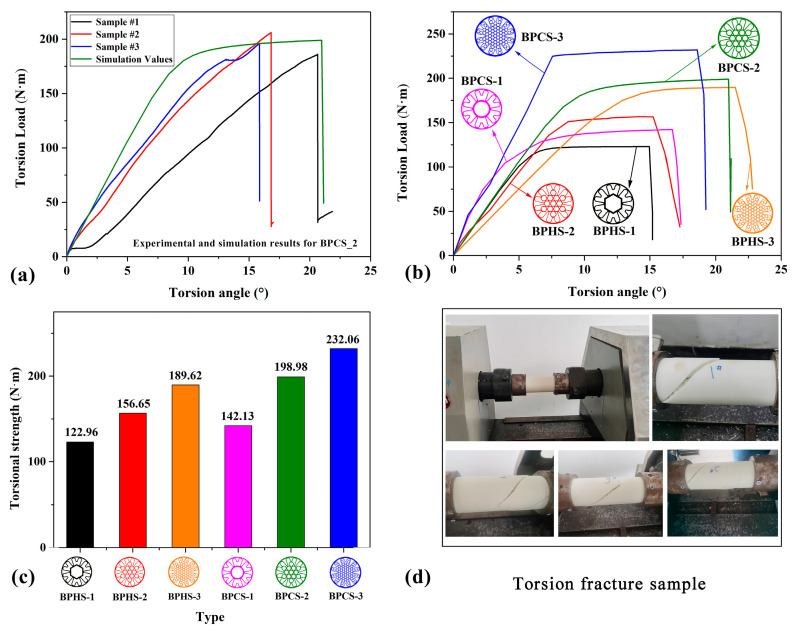
Torsional characteristics of the various proposed structures. (**a**) Comparison of the torsional strength between experimental and simulated outcomes for BPCS_2. (**b**) Simulation curves of torsion load angle for the proposed structures. (**c**) The maximum torsional load. (**d**) The torsion property test.

The torsional properties of the BPSs are clearly illustrated in Figure 7. Figure 7a presents a comparison between the experimental results and simulation results for the torsional properties of BPCS_2. It can be observed that the torsion load increases with the increment of the torsion angle in all three curves until reaching the maximum value. Subsequently, the thin tube undergoes failure, resulting in a sharp drop in torsion load. The trends of these curves demonstrate a similar pattern. Additionally, it is apparent that the maximum torsional load corresponding to failure in the three curves is quite close. By examining the detailed torsional load data for BPCS_2 in Table 5, it can be concluded that the actual maximum torsional load of the three samples has an average difference of 1.5% compared to the maximum torsional load value obtained from the simulation experiment. This observation suggests that the finite element method (FEM) model effectively and accurately reflects the experimental data on thin tube torsion.

Figure 7b depicts the torsional load–torsion angle simulation curves for the different proposed structures. It can be observed that all curves exhibit a consistent trend, with the torsion load increasing as the torsion angle rises. Eventually, the thin tube undergoes failure, leading to a sharp decline in the torsion load. Notably, BPCS 3 displays the highest maximum torsional load and exhibits superior torsional properties. On the other hand, BPHS_1 demonstrates the lowest maximum torsional load and poorer torsional resistance. The maximum torsional loads of BPHS_1, BPHS_2, and BPCS_1 are similar, indicating comparable torsional characteristics.

Furthermore, Figure 7c clearly illustrates the maximum torsional loads for the different proposed structures. It is evident that BPCS_3 exhibits the highest maximum torsional load, measuring 232.06 N·m. Conversely, BPHS_1 exhibits the lowest maximum torsional load, measuring 122.96 N·m. BPHS_3 and BPCS_2 are closely situated, with BPCS_2 surpassing BPHS_3 by 4.94%. When comparing the maximum torsional loads of BPHS_1, BPHS_2, and BPHS_3, it is evident that BPHS_3 exhibits the highest maximum torsional load at 189.62 N·m. Conversely, BPHS_1 has the lowest maximum torsional load of 122.96 N·m. Furthermore, the maximum torsional load of BPHS_2 is 27.40% higher than that of BPHS_1, while BPHS_3 surpasses BPHS_2 by 21.05%. The results indicate that the maximum torsional load increases significantly with the number of hexagonal structures filled within the thin tube. This suggests that filling the thin tube with these structures can notably enhance its torsional properties. Similarly, when comparing BPCS_1, BPCS_2, and BPCS_3 in terms of maximum torsional load, there is a significant increase as the number of circular structures filled within the thin tube rises. Specifically, BPCS_1 displays the lowest maximum torsional load at 142.13 N·m, while BPCS_3 demonstrates the highest at 232.06 N·m. The maximum torsional load of BPCS_2 is 40.00% higher than that of BPCS_1, and BPCS_3 exceeds BPCS_2 by 16.62%. These findings suggest that filling the thin tube with circular structures can also substantially enhance its torsional resistance properties. Additionally, when comparing BPHS_1 and BPCS_1, BPCS_1 surpasses BPHS_1 by 15.59%. Similarly, BPCS_2 exceeds BPHS_2 by 27.02%.

Figure 7d illustrates the actual torsional performance test, revealing clear cracks as the thin tube undergoes twisting and eventual destruction. Table 7 presents the torsion experimental data for all proposed structures. Analysis of Figure 7 indicates that BPHS_1 exhibits the poorest performance among all thin tubes, whereas BPCS_3 shows the best performance. Moreover, according to Table 7, BPHS_1 demonstrates the lowest LWN-T value of 0.99 (N·m)/g, while BPCS_3 displays the highest LWN-T value of 1.88 (N·m)/g. These values align with the analysis of the thin tube’s torsional properties.

### 3.5. Crashworthiness Behaviors

**Figure 8:**
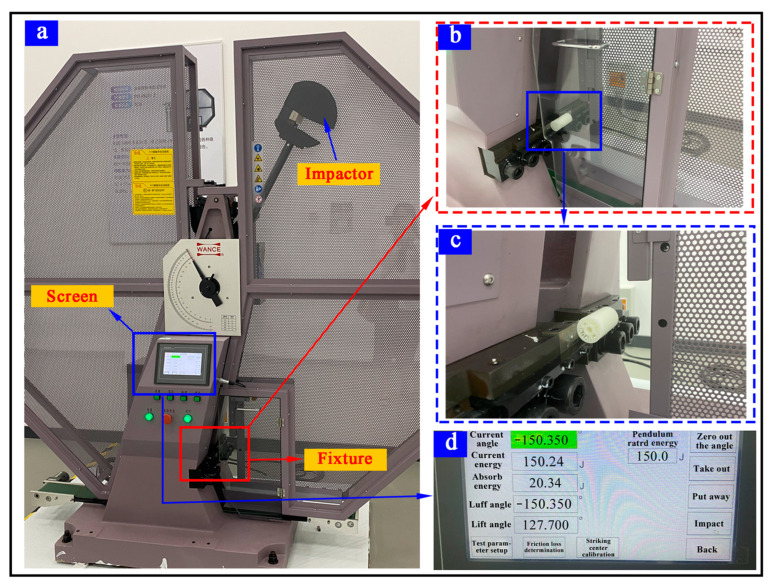
Pendulum impact testing machine. (**a**) Pendulum tester; (**b**) test fixture; (**c**) an enlarged view of (**b**); (**d**) testing user interface.

The crashworthiness of BPSs was investigated using a pendulum impact testing machine (PIT452D-2, Shenzhen Wance, Shenzhen, China), as shown in Figure 8. The maximum impact energy of the pendulum impact testing machine was set at 150 J, while the impact velocity was maintained at 5.24 m per second. During the testing, the specimens were exposed to a negative swing angle of −150.350° followed by a positive swing angle of 127.70°. The impact data of samples for each BPS are presented in Table 8.

Table 8 presents the results of the impact test, which was conducted separately for each thin tube structure using three samples to reduce unexpected errors and ensure result accuracy. It can be observed that BPHS_3 and BPCS_3 have higher SEA values, with BPCS_3 slightly outperforming BPHS_3. This suggests a strong energy absorption capacity for both structures, but BPCS_3 exhibits superior energy absorption compared to BPHS_3. Among the tested structures, BPCS_3 demonstrates the highest energy absorption capacity. On the other hand, BPHS_1 and BPCS_1 exhibit low SEA values, with BPCS_1 slightly surpassing BPHS_1. This indicates weak energy absorption capacity for both structures, where BPCS_1 performs better than BPHS_1. Notably, BPHS_1 shows the weakest energy absorption capability. Furthermore, an increasing trend in SEA values is observed for BPHS_1, BPHS_2, and BPHS_3. Similarly, BPCS_1, BPCS_2, and BPCS_3 display a gradual rise in SEA values. This trend is related to the number of filled polygonal structures present inside the thin tubes. Additionally, it is worth mentioning that the average SEA value of BPHSs is lower than the corresponding BPCSs’ average SEA value. This difference stems from the distinct types of polygonal structures employed in filling the thin tubes, with hexagonal structures used for BPHSs and circular structures used for BPCSs.

To further investigate the crashworthiness properties of thin-walled tubes, we further processed the data in Table 8 to Table 9, which more intuitively demonstrates the SEA values of BPHSs and BPCSs. It can be found that the SEA values of BPHS_3 and BPCS_3 are higher, where the mean SEA value of BPCS_3 is 13.52% higher than that of BPHS_3. Furthermore, the mean SEA value of BPCS_1 is 31.6% higher than BPHS_1, and the mean SEA value of BPCS_2 is 7.70% higher than BPHS_2. The mean SEA values of BPCSs are all higher than the mean SEA values of the corresponding BPHSs, due to the circular structure filled in BPCSs and the hexagonal structure filled in BPHSs. It shows that filling the circular structure in the thin tube is more effective than filling the hexagonal structure in improving the energy absorption capacity of the thin tube. The comparison of the mean SEA values of BPHS_1, BPHS_2, and BPHS_3 shows that BPHS_1 has the lowest mean SEA value of 353.76 J/kg, and BPHS_3 has the highest mean SEA value, at 810.27 J/kg. Moreover, the mean SEA value of BPHS_2 is 98.17% higher than that of BPHS_1, and the mean SEA value of BPHS_3 is 15.58% higher than that of BPHS_2. This is because BPHS_1 has the least number of internally filled hexagonal structures and BPHS_3, indicating that increasing the number of filled polygon structures in the thin tube can effectively improve the energy absorption capacity of the thin tube. Furthermore, a comparison of the mean SEA values of BPCS_1, BPCS_2, and BPCS_3 shows that BPCS_1 has the lowest mean SEA value, which is 465.64 J/kg, and BPCS_3 has the highest mean SEA value of 919.82 J/kg. Moreover, the mean SEA value of BPCS_2 is 62.14% higher than that of BPCS_1, and the mean SEA value of BPCS_3 is 21.83% higher than that of BPCS_2. This is due to the least number of circular structures inside BPCS_1 and BPCS_3, which also indicates that increasing the number of filled polygonal structures in the thin tube can effectively improve the energy absorption capacity of the thin tube. BPCS_3 had the highest SEA value and the best energy absorption capacity in the shock test.

**Figure 9:**
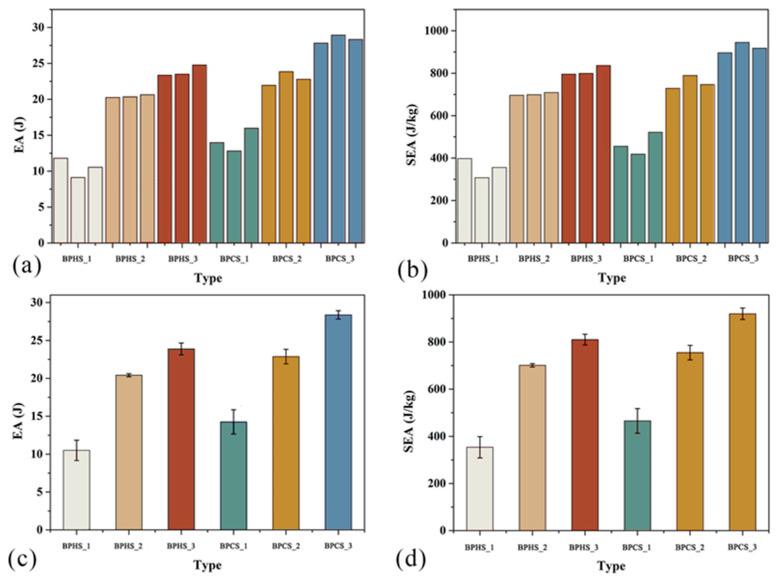
Results of impact test. Panels (**a**,**b**) display the values of each BS sample for EA and SEA. Panels (**c**,**d**) show the average values of EA and SEA for each BPS sample.

Figure 9 bar chart visually represents the impact of experimental data. Figure 9a displays the EA values for all BHPSs and BPCSs, while Figure 9b shows the SEA values for these samples. Observing the charts, it is evident that the three samples of BHPS_1 and BPCS_1 exhibit lower EA values, which correspond to lower SEA values as well. On the other hand, the three samples of BPHS_3 and BPCS_3 display higher EA values, indicating higher SEA values as well. Further analysis of the data led to the creation of Figure 9c,d. Figure 9c presents the average Bed EA values for all BHPSs and BPCSs, whereas Figure 9d showcases the mean SEA values for these samples. Upon closer examination, it can be observed that BHPS_1 has the lowest mean EA value, accompanied by a lower SEA value, indicating poor energy absorption characteristics. Conversely, BPCS_3 exhibits the highest mean EA value, along with a higher corresponding SEA value, suggesting better energy absorption characteristics. Additionally, the EA and SEA values of BPHS_1, BPHS_2, and BPHS_3 show an increasing trend. Similarly, the EA and SEA values of BPCS_1, BPCS_2, and BPCS_3 gradually increase as well. Furthermore, it is worth noting that the EA and SEA values of all BHPSs are slightly lower than the EA and SEA values of the corresponding BPCSs. 

### 3.6. Optimization Based on Response Surface Methodology

The response surface methodology is employed for optimization purposes. The focus of the optimization is on enhancing the performance of the BPHS_1 component, with specified boundary and loading conditions including restrictions on 6 degrees of freedom at one end, coupled with the application of a pressure of 10 kN and a torque of 100 N·m at the other end. The optimization objectives encompass minimizing the structural mass, maximum strain, and maximum deformation while adhering to the constraint that the maximum stress must not exceed the allowable limit. The mathematical model utilized for optimization purposes is HPHS_1 which serves as the key parameter of interest for optimization, and minimizing structural mass, maximum strain, and maximum deformation are the primary optimization objectives, all while ensuring that the maximum stress remains within acceptable levels.

(7) $$\left\{\begin{array}{c} x_{i}^{\left(t\right)}\leq x_{i}\leq x_{i}^{\left(m\right)}\\ \sigma_{\max}\leq \left[\sigma \right]\\ \min\left(\mathrm{Strain}\right)\\ \min\left(\mathrm{Mass}\right)\\ \min\left(\mathrm{Deformation}\right) \end{array}\right.$$

where $x_{i}^{\left(t\right)}$—lower bound of design variables; $x_{i}^{\left(m\right)}$—upper bound of design variables; $\sigma_{\max}$—maximum stress; $\left[\sigma \right]$—allowable stress.

**Figure 10:**
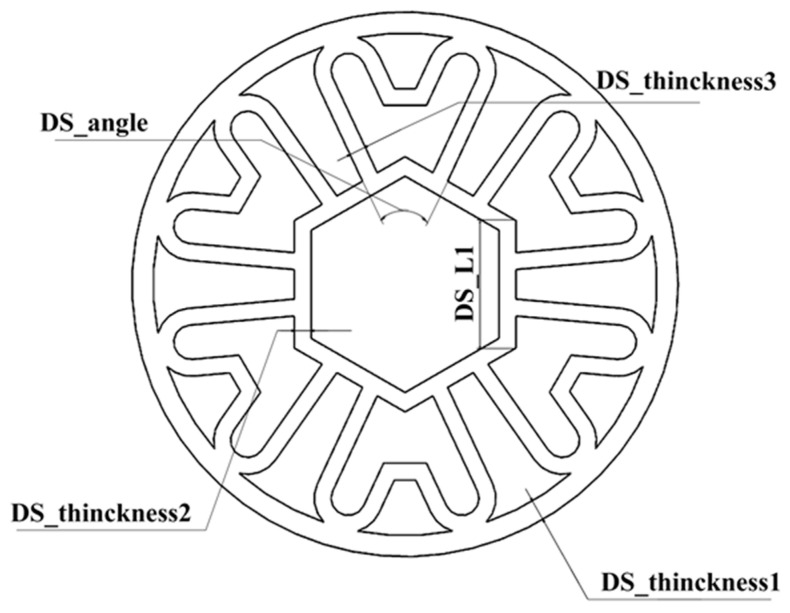
Design variables dimensional parameters.

Prior to optimizing the design, a parametric model of the bionic tube structure was created and validated using ANSYS. Five key structural dimensions were identified as design variables, as illustrated in Figure 10. The permissible range of variation for each variable was set at ±10%, with the specific value range detailed in Table 10.

**Figure 11:**
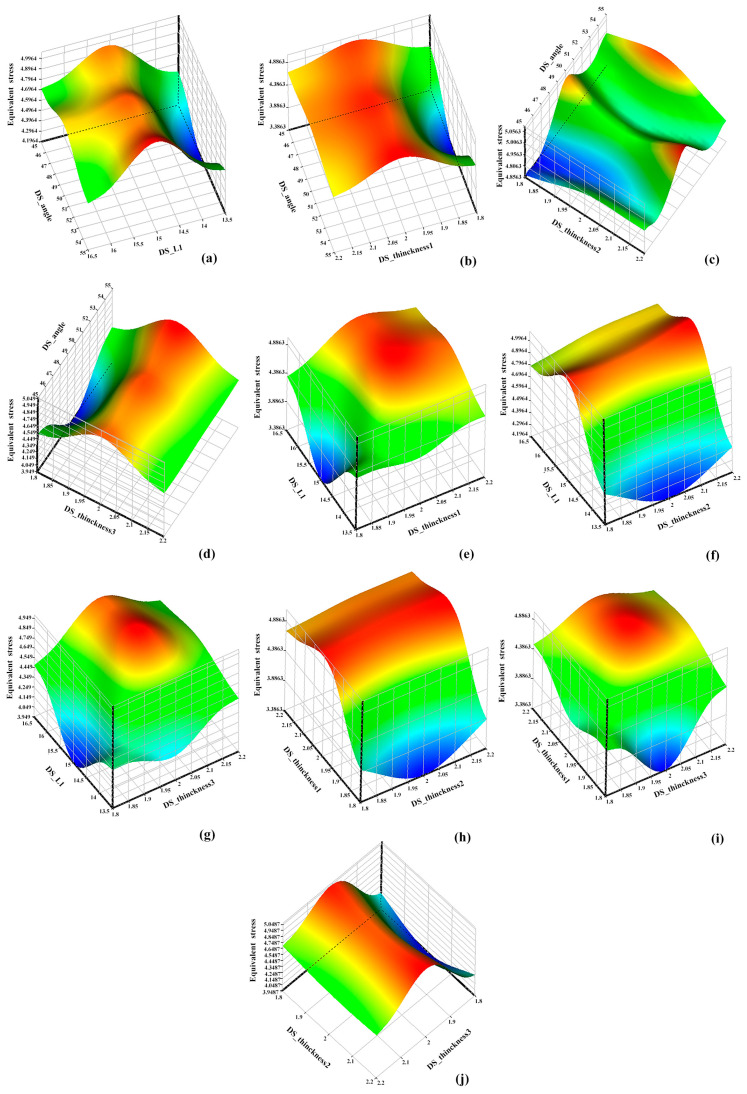
Response surfaces of structural parameters and equivalent stress. (**a**) Angle-L1-equivalent stress; (**b**) Angle-Thinckness1-equivalent stress; (**c**) Angle-Thinckness2-equivalent stress; (**d**) Angle-Thinckness3-equivalent stress; (**e**) L1-Thinckness1-equivalent stress; (**f**) Thinckness1-Thinckness2-equivalent stress; (**g**) L1-Thinckness3-equivalent stress; (**h**) Thinckness1-Thinckness2-equivalent stress; (**i**) Thinckness1-Thinckness3-equivalent stress; (**j**) Thinckness2-Thinckness3-equivalent stress.

**Figure 12:**
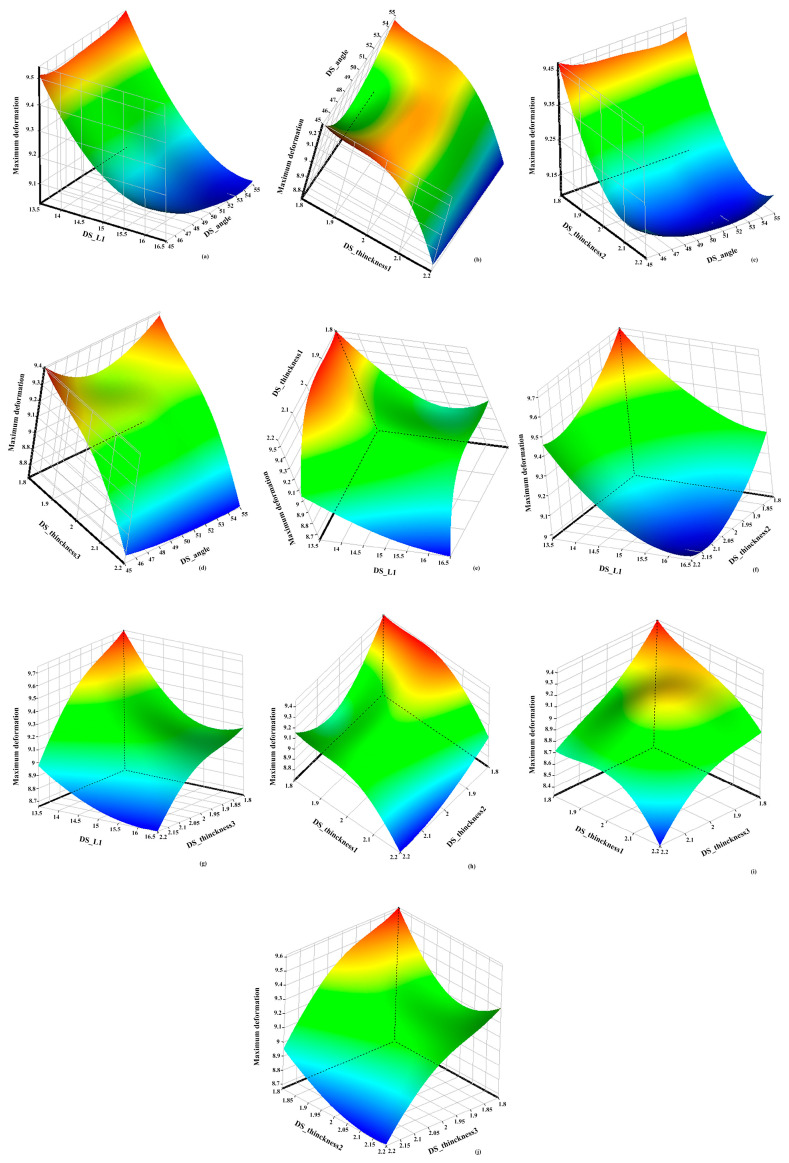
Response surfaces of structural parameters and maximum deformation. (**a**) Angle-L1-maximum deformation; (**b**) Angle-Thinckness1-maximum deformation; (**c**) Angle-Thinckness2-maximum deformation; (**d**) Angle-Thinckness3-maximum deformation; (**e**) L1-Thinckness1-maximum deformation; (**f**) Thinckness1-Thinckness2-maximum deformation; (**g**) L1-Thinckness3-maximum deformation; (**h**) Thinckness1-Thinckness2-maximum deformation; (**i**) Thinckness1-Thinckness3-maximum deformation; (**j**) Thinckness2-Thinckness3-maximum deformation.

By analyzing the simulation calculation results of the sample points, the response surfaces of the design variables and the target function were determined. Figure 11 and Figure 12 illustrate the impact of these parameters on equivalent strain and maximum deformation. 

A response surface analysis was conducted involving the collection of 5000 samples using a screening method. Subsequently, a multi-objective genetic optimization algorithm (MOGA) was utilized to identify the optimal solution. Through Figure 11 and Figure 12, the objective function can be observed while keeping other design variables constant. Specifically, from Figure 11a–d, it can be observed that when maintaining the DS_angle constant, the equivalent stress initially increases and then decreases with an increase in DS_L1 and DS_Thickness, indicating a significant influence of these two design variables on equivalent stress. Furthermore, Figure 11e–g reveal that when keeping DS_L1 constant, the variation in DS_Thickness leads to changes in the response surface, with the equivalent stress increasing to a maximum value in cases (e) and (g) as DS_Thickness increases, while in case (f), the equivalent stress reaches its maximum value at the initial stage of DS_Thickness. Figure 11h–j consider the impact of two different DS_Thickness on equivalent stress. Additionally, Figure 12a–d demonstrate that, with DS_angle held constant, the maximum deformation decreases gradually with increasing DS_L1 and DS_Thickness. Moreover, in Figure 12e–g, it is observed that when keeping DS_L1 constant, the maximum deformation decreases gradually as DS_Thickness increases. Particularly, the slope of the maximum deformation related to DS_Thickness1 decreases faster, indicating a higher sensitivity of maximum deformation to DS_Thickness1. Figure 12h–j simultaneously consider the impact of two different DS_Thickness on maximum deformation, showing that the location of the maximum deformation remains the same and the trend of change is similar, decreasing with an increase in DS_Thickness.

This resulted in the identification of three distinct sets of alternative solutions, each offering unique benefits and trade-offs. The results of this analysis are summarized in Table 11. This comprehensive approach allowed for a thorough exploration of the design space and enabled the selection of solutions that best met the specified objectives.

In the analysis of variances, *R*^2^, adjusted *R*^2^ (*R*^2^*_adj_*), and root mean square error (*RMSE*) are employed to evaluate the fitting accuracy, which can be calculated as follows:

(8) $$R^{2}=1-\frac{SSE}{SST}$$

(9) $$RMSE=\sqrt{\frac{SSE}{M-p-1}}$$

(10) $$R_{adj}^{2}=1-\left(1-R^{2}\right)\frac{M-1}{M-p-1}$$

(11) F=SST−SSEpSSEM−p−1

The number of sample points, the sum of squared errors (*SSE*), and the total sum of squares (*SST*) are used to calculate the fitting accuracy in the analysis of variances, with the number of non-constant terms in the RS model denoted as *p*. The calculations for *SSE* and *SST* are as follows:

(12) $$SSE={\sum_{i=1}^{n}\left(y_{i}-\overset{\wedge }{y_{i}}\right)}^{2}$$

(13) $$SST={\sum_{i=1}^{n}\left(y_{i}-\bar{y_{i}}\right)}^{2}$$

where *y_i_* represents the FEA result, $\overset{\wedge }{y_{i}}$ represents the RS approximation value, and $\bar{y_{i}}$ is the mean value of *y_i_*. 

**Figure 13:**
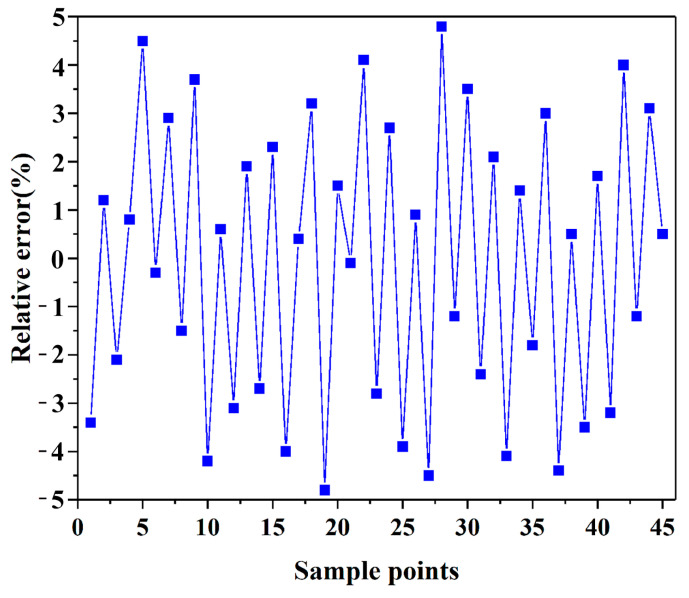
Relative errors of response surface function. The blue line represents the fluctuation curve of sample points’ relative errors. The squares represent the sample points.

In the context of a precision inspection, the values of *R*^2^ and *R*^2^*_adj_* are both above 0.97, affirming the high accuracy of the response surface model in the fitting. This suggests that the model is capable of meeting the precision requirements for parameter optimization of the proposed structure. The error assessments for shape optimization using the response surface are provided in Table 12. The maximum relative errors fall within a 5% margin (see Figure 13), underscoring the strong accuracy of the RS models involved in this study. The comparison presented in Table 13 reveals notable improvements in various output parameters between the original solution and the optimized solutions I, II, and III. Specifically, when considering maximum deformation, the optimized solutions I, II, and III show a decrease of 26.97%, 26.97%, and 26.84% compared to the original solution, respectively. In terms of maximum strain, the optimized solutions I, II, and III demonstrate substantial reductions of 85.56%, 85.06%, and 84.78%, respectively. Furthermore, there is a consistent decrease in mass across the optimized solutions, with reductions of 3.38%, 3.33%, and 3.33% for solutions I, II, and III, respectively, when compared to the original solution. Based on the analysis results, it is clear that optimized solution I stands out as the best choice due to its lower mass and strain compared to solutions II and III. It is evident from the data that the optimized solutions have significantly enhanced performance across all parameters, showcasing the effectiveness and consistency of the optimization process in improving system efficiency and effectiveness.

## 4. Conclusions

Valuable insights into the nanomechanical and macro-mechanical properties of porcupine quills were provided by this study. The hardness and elastic modulus at the nanoscale were evaluated through nanoindentation testing, while the macro-scale mechanical response was assessed through tensile testing using a universal testing machine. Furthermore, an optical microscope was utilized to observe the micro- and macro-structural features of the quills. Based on the structural analysis, various biomimetic porous structures (BPSs) inspired by the porcupine quill morphology were designed. These structures underwent compression, torsion, and impact performance studies to investigate their mechanical behavior under different loading conditions. The main findings can be summarized as follows:

(1) The porcupine quill exhibits significant variations in mechanical properties among its different regions. The upper and lower epidermis have higher elastic moduli (8.13 ± 0.05 GPa and 7.71 ± 0.14 GPa, respectively) compared to other regions, while the mid-dermis shows intermediate values (7.16 ± 0.10 GPa). Additionally, the elastic modulus at the center of each cross section is relatively lower than that of the surrounding area, with values of 3.93 ± 0.86 GPa for the upper cross section, 3.48 ± 0.46 GPa for the mid-cross section, and 2.31 ± 0.71 GPa for the lower cross section.
(2) The hardness values follow a similar trend to the elastic modulus in different regions of the porcupine quill. The upper and mid-epidermis exhibit higher hardness values (0.38 ± 0.02 GPa and 0.34 ± 0.01 GPa, respectively) compared to the lower epidermis (0.36 ± 0.01 GPa). Furthermore, significant differences in hardness are observed among the different regions of the quill, with values of 0.16 ± 0.09 GPa for the upper cross section, 0.16 ± 0.12 GPa for the mid-cross section, and 0.06 ± 0.01 GPa for the lower cross section.
(3) The porcupine quill demonstrates high tensile strength, with a value of 248.95 ± 35.88 N, and its modulus of elasticity results of 30.28 ± 10.98 MPa.
(4) The results indicate that different types of BPSs exhibit similar compression performance. BPHS_1 demonstrated the highest maximum compression load (54,989 N), followed by BPHS_2 and BPHS_3. Similarly, BPCS_1 exhibited the highest maximum compression load (55,215 N), followed by BPCS_2 and BPCS_3. These findings suggest that the incorporation of hexagons and circles has minimal impact on compression performance.
(5) BPCS_3 exhibited the highest maximum torsional load at 232.06 N·m, while BPHS_1 had the lowest at 122.96 N·m. BPCS_2 surpassed BPHS_3 by 4.94%, indicating superior torsional properties for BPCS_2. The maximum torsional load increased significantly with the number of hexagonal or circular structures filled within the thin tube, suggesting significant improvement in torsional resistance properties.
(6) The overall crashworthiness of the structures improved as the number of filled polygonal and circular structures increased, with BPCS_3 demonstrating the highest average energy absorption (28.37 J) and specific energy absorption (919.82 J/kg), indicating superior performance compared to other structures. Conversely, BPHS_1 exhibited the lowest average energy absorption (10.49 J) and specific energy absorption (353.76 J/kg), reflecting its poor energy absorption capability.
(7) Based on the response surface optimization method proposed, solution I achieved reductions of 3.38% in mass, 26.97% in maximum deformation, and 85.56% in maximum strain compared to the original solution, emphasizing its superior performance in system efficiency and effectiveness.

In conclusion, the BPS structures were designed to mimic the gradient properties of porcupine quills, including their multi-layered microstructure, cell arrangement characteristics, and porous structure, to enhance mechanical properties and impact resistance. Among the BPS structures, BPCS_3 stands out as the optimal choice for applications requiring lightweight design, such as aerospace and automotive industries, robotic arms, and drive shafts. Despite potential manufacturing cost limitations compared to simple circular tube structures, BPCS_3 excels in lightweight, high strength, and energy absorption capabilities. Its customizable structures, energy absorption, and acoustic/thermal insulation performance make it a versatile material for various applications needing lightweight, high-strength design with exceptional mechanical properties and impact resistance.

## Figures and Tables

**Table 1:** Material properties of R4600 resin material.

Material Name	R4600 Resin Material
Density (g/cm^3^)	1.3
Young’s modulus (MPa)	2600
Poisson’s ratio	0.42
Elongation at break	10%
Tensile strength (MPa)	47

**Table 2:** Modulus of the different regions in porcupine quill.

Type	Modulus at Max Load (GPa)					
	Upper Epidermis	Upper Cross Section	Mid-Dermis	Mid-Cross Section	Lower Epidermis	Lower Cross Section
Sample #1	8.15	5.37	7.28	3.75	7.65	2.05
Sample #2	8.05	3.21	7.00	2.38	8.06	3.22
Sample #3	8.15	2.76	7.07	3.49	7.66	2.07
Sample #4	8.15	4.51	7.10	3.90	7.67	1.39
Sample #5	8.18	4.43	7.28	3.63	7.65	3.13
Sample #6	8.17	4.11	7.17	3.53	7.65	2.16
Sample #7	8.08	3.96	7.21	3.59	7.66	2.99
Sample #8	8.17	3.12	7.19	3.60	7.67	1.51
Mean ± SD	8.13 ± 0.05	3.93 ± 0.86	7.16 ± 0.10	3.48 ± 0.46	7.71 ± 0.14	2.31 ± 0.71

**Table 3:** Hardness of the different regions in porcupine quill.

Type	Hardness at Max Load (GPa)					
	Upper Epidermis	Upper Cross Section	Mid-Dermis	Mid-Cross Section	Lower Epidermis	Lower Cross Section
Sample #1	0.40	0.29	0.37	0.27	0.34	0.07
Sample #2	0.38	0.09	0.33	0.05	0.38	0.08
Sample #3	0.39	0.09	0.33	0.06	0.34	0.04
Sample #4	0.38	0.26	0.34	0.35	0.35	0.05
Sample #5	0.35	0.20	0.35	0.25	0.37	0.07
Sample #6	0.36	0.15	0.34	0.06	0.35	0.07
Sample #7	0.39	0.12	0.34	0.15	0.36	0.07
Sample #8	0.36	0.10	0.34	0.09	0.37	0.05
Mean ± SD	0.38 ± 0.02	0.16 ± 0.09	0.34 ± 0.01	0.16 ± 0.12	0.36 ± 0.01	0.06 ± 0.01

**Table 4:** Fit curves.

Fit Curves	Intercept		Slope		Statistics
	Value	Standard Error	Value	Standard Error	Adj. R-Square
Sample #1	8.71	0.12	48.94	0.55	0.96
Sample #2	9.97	0.13	26.43	0.27	0.93
Sample #3	8.20	0.11	30.78	0.24	0.96
Sample #4	5.57	0.08	21.76	0.17	1.0
Sample #5	6.25	0.10	23.48	0.21	0.94

**Table 5:** ANOVA summary table.

Sample Number	Model SS	Error SS	F Value	Prob > F
Sample #1	9268.17	383.88	8039.67	0
Sample #2	27,056.00	2071.29	9404.94	0
Sample #3	36,697.23	1625.12	16,258.50	0
Sample #4	18,337.43	767.25	17,208.20	0
Sample #5	21,348.99	1286.28	11,950.13	0

**Table 6:** Comparison between compression data from experiments and simulations.

Type	Simulation/Test Values	Mass (g)	Maximum Compressive Load (kN)	LWN-C (N/g)
BPHS_1	Simulation values	123.71	54.99	444.51
BPHS_2	Simulation values	123.71	53.59	433.19
BPHS_3	Simulation values	123.71	54.05	436.91
BPCS_1	Simulation values	123.71	55.22	446.37
BPCS_2	Test sample #1	123.88	53.27	430.01
	Test sample #2	124.67	53.57	429.69
	Test sample #3	123.32	53.48	433.67
	Simulation values	123.71	53.67	433.84
BPCS_3	Simulation values	123.71	55.25	446.61

**Table 7:** Comparison of experimental and simulated torsion data.

Type	Simulation/Test Values	Mass (g)	Maximum Torsional Load (N·m)	LWN-T (N·m)/g
BPHS_1	Simulation values	123.71	122.96	0.99
BPHS_2	Simulation values	123.71	156.65	1.27
BPHS_3	Simulation values	123.71	189.62	1.53
BPCS_1	Simulation values	123.71	142.13	1.15
BPCS_2	Test sample #1	123.26	186.01	1.51
	Test sample #2	123.75	205.99	1.66
	Test sample #3	123.91	196.00	1.58
	Simulation values	123.71	198.98	1.61
BPCS_3	Simulation values	123.71	232.06	1.88

**Table 8:** Results of impact test.

Types	Samples	Mass (g)	EA (J)	SEA (J/kg)
BPHS_1	#1	29.66	11.8	397.84
	#2	29.68	9.13	307.61
	#3	29.62	10.54	355.84
BPHS_2	#1	29.10	20.25	695.88
	#2	29.13	20.34	698.25
	#3	29.11	20.64	709.03
BPHS_3	#1	29.37	23.36	795.37
	#2	29.42	23.51	799.12
	#3	29.63	24.78	836.31
BPCS_1	#1	30.64	13.97	455.94
	#2	30.60	12.82	418.95
	#3	30.63	15.99	522.04
BPCS_2	#1	30.12	21.96	729.08
	#2	30.22	23.85	789.21
	#3	30.52	22.79	746.72
BPCS_3	#1	31.05	27.83	896.30
	#2	30.62	28.94	945.13
	#3	30.86	28.33	918.02

**Table 9:** Crashworthiness indicators of each proposed structure (Mean ± SD).

Types	EA (J)	SEA (J/kg)
BPHS_1	10.49 ± 1.34	353.76 ± 45.15
BPHS_2	20.41 ± 0.20	701.05 ± 7.00
BPHS_3	23.88 ± 0.78	810.27 ± 22.63
BPCS_1	14.26 ± 1.60	465.64 ± 52.23
BPCS_2	22.87 ± 0.95	755.00 ± 30.91
BPCS_3	28.37 ± 0.56	919.82 ± 24.46

**Table 10:** Design variable constraints.

Design Variables	Initial Value	Lower Bound	Upper Bound
DS_angle (°)	50°	45°	55°
DS_L1 (mm)	15	13.5	16.5
DS_Thinckness 1 (mm)	2	1.8	2.2
DS_Thinckness 2 (mm)	2	1.8	2.2
DS_Thinckness 3 (mm)	2	1.8	2.2

**Table 11:** Candidate solutions.

Input and Output Parameters	Optimization Solutions I	Optimization Solutions II	Optimization Solutions III
DS_angle (°)	54.988°	54.948°	54.903°
DS_L1 (mm)	16.490	16.496	16.496
DS_Thinckness 1 (mm)	2.197	2.200	2.197
DS_Thinckness 2 (mm)	2.200	2.195	2.199
DS_Thinckness 3 (mm)	1.801	1.802	1.802
Maximum deformation (mm)	5.47 × 10^−4^	5.47 × 10^−4^	5.48 × 10^−4^
Maximum strain	2.60 × 10^−3^	2.69 × 10^−3^	2.74 × 10^−3^
Mass (kg)	0.1656	0.1657	0.1657

**Table 12:** Error measures of the RS for shape optimization.

	*R* ^2^	*R* ^2^ *_adj_*	*RMSE*
Mass	0.99	0.98	0.19
Maximum deformation	0.98	0.97	0.18
Maximum strain	0.99	0.99	0.19

**Table 13:** Performance comparison between the original and optimized solutions.

Output Parameters	Original Solution	Optimization Solutions I	Optimization Solutions II	Optimization Solutions III
Maximum deformation (mm)	7.49 × 10^−4^	5.47 × 10^−4^	5.47 × 10^−4^	5.48 × 10^−4^
	/	−26.97%	−26.97%	−26.84%
Maximum strain	1.8 × 10^−2^	2.60 × 10^−3^	2.69 × 10^−3^	2.74 × 10^−3^
	/	−85.56%	−85.06%	−84.78%
Mass (kg)	0.1714	0.1656	0.1657	0.1657
	/	−3.38%	−3.33%	−3.33%

Note: Table 12—represents the percentage reduction compared to the original solution.

## Data Availability

Data are contained within the article.
